# Interventions within the Scope of Occupational Therapy in the Hospital Discharge Process Post-Stroke: A Systematic Review

**DOI:** 10.3390/healthcare10091645

**Published:** 2022-08-29

**Authors:** Patricia García-Pérez, José Pablo Lara, María del Carmen Rodríguez-Martínez, Carlos de la Cruz-Cosme

**Affiliations:** 1 Faculty of Medicine, University of Málaga, Blvr. Louis Pasteur, 32, 29010 Málaga, Spain; 2 Occupational Therapy Department, Virgen de la Victoria University Hospital, Servicio Andaluz de Salud (SAS), 29010 Málaga, Spain; 3 Brain Health Unit, CIMES, 29010 Málaga, Spain; 4 Malaga Biomedical Research Institute (IBIMA), 29010 Málaga, Spain; 5 Department of Physiotherapy, Faculty of Health Sciences, University of Málaga, C/Arquitecto Francisco Peñalosa 3, 29071 Málaga, Spain; 6 Neurology Department, Virgen de la Victoria University Hospital, 29010 Málaga, Spain

**Keywords:** stroke, rehabilitation, patient discharge, caregivers, occupational therapy

## Abstract

Stroke is one of the main causes of disability in adulthood. Its rehabilitation is a complex process that requires a multidisciplinary team of specialised professionals. The main goal of this review was to determine the impact of occupational therapy intervention post-stroke on the home discharge process. A systematic search was carried out of the following databases: Pubmed, Web of Science, PsycINFO, Scopus, Otseeker, and Dialnet. A screening was performed taking into account the type of article, including exclusively RCT, and type of intervention, only including interventions within the scope of occupational therapy that take place during the process of hospital discharge post-stroke. A total of 2285 citations were identified in the search from which 13 articles met the inclusion criteria. Comparisons of the groups indicated that early occupational therapy intervention during the process of hospital discharge can be effective in terms of functional recovery and can lead to the caregiver seeing improvements in self-efficacy and fatigue. In addition, the inclusion of a caregiver in the intervention influences the patient’s adherence to treatment, leading to a reduction in the cost of treatment and rehabilitation.

## 1. Introduction

It is estimated that two out of every three people who suffer a cerebrovascular accident (CVA) have sequelae that affect their quality of life and CVA is the main cause of severe disability in adulthood [1,2,3]. Stroke rehabilitation is a complex process that requires a multidisciplinary team of qualified professionals and constitutes the most effective treatment to reduce functional deficits. The rehabilitation intervention must be tailored to the patient, immediate, frequent, and intensive [4,5]. Nowadays, practices used in rehabilitation mostly consist of contributions from the international stroke rehabilitation guidelines [6]. Nevertheless, the treatment for people with stroke often varies considerably within a given country, with the clearest and most common division being between urban and rural areas [7]. In Spain, stroke represents the second leading cause of death and the first cause of disability in adulthood, creating a significant healthcare and social burden [8]. According to data, the annual incidence of stroke in Spain is 187 to 252 per 100,000 inhabitants [9]. A recent study shows that mortality rates in Spain from CVA have slowed, are stagnant, and have even been reversed, showing a change in the prognosis of stroke in recent years, which is similar to global trends. Furthermore, survival and functional outcomes have also improved after the introduction of a new model of care based on stroke units [10,11,12,13]. Although the exact start time and intensity of stroke rehabilitation are controversial, evidence shows that it is beneficial to start the process as soon as the patient is ready and able to tolerate it. Early mobilisation should be undertaken carefully and requires a multidisciplinary team of specialised professionals in stroke or intensive care units [14,15].

A study recently published by The Kings College of London in collaboration with the European Stroke Alliance (SAFE) aimed to determine the impact of stroke and how the need for acute care and follow-up was being met in each country in the EU [16]. Based on this data, a multidisciplinary assessment in the acute phase in the first 48 h was shown to be ideal, but these quality standards are not always achieved. Access to occupational therapy (OT), physiotherapy, and speech and language therapy (SALT) in the neurorehabilitation process is another example of the different experiences depending on the region [17]. Scientific data regarding the transition from hospital to home as part of a structured discharge planning model has shown that these programs vary in structure and focus although they include common characteristics such as the time of admission to hospital, patient and caregiver involvement throughout the process, and good communication [18]. It has also been shown that home treatment is effective and that the results are maintained beyond 6 months, postulating this type of service for when the accessibility of outpatient services may be a challenge [19,20,21,22,23,24]. Such evidence has led countries such as Sweden and the United Kingdom to form early supported discharge (ESD) teams, which consist of a consultation with a multidisciplinary team that can ensure the coordination of care from multidisciplinary hospital stroke teams to community teams, which has also shown to significantly improve the quality of life and patient satisfaction and to reduce caregiver stress [25,26,27]. In addition, these services have been shown to offer patients a greater chance of regaining independence in their daily activities and there is evidence of their cost-effectiveness [19,21,28,29,30]. Around the world they have been reported to reduce the frequency of healthcare use and change the focus of expenditure, resulting in more spending on OT, physiotherapy, and SALT in the community than in hospitals [20]. The ESD team should comprise the following professionals with expertise in stroke rehabilitation: consultant physicians, nurses, physiotherapists, occupational therapists, speech and language therapists, clinical psychologists, rehabilitation assistants, and social workers [31]. However, not all European countries have embraced these types of services and have not even considered the burden on the occupational therapist alone to support post-stroke hospital discharge patients. Despite the short length of hospitalisation in the acute care setting, occupational therapist practitioners play an essential role in a successful rehabilitation process and are also responsible for assessing the patient’s home environment and recommending safety modifications and medical equipment to support functional performance after discharge [32].

OT constitutes a patient-centred profession that influences all sensory, motor, cognitive, and affective functions through meaningful activities with the aim of reducing limitations and enhancing skills and plays a very important role in the discharge process. It takes into account the importance of the natural context, which emphasises the need to carry out home visits that can ensure the transfer of knowledge as well as adaptations to the environment [33,34]. Making an efficient transition of care has the potential to promote integrated and coordinated care, maximise functional recovery, and support a stroke patient’s return to the natural context. Discharge planning with the support of the occupational therapist can facilitate good community integration and participation, which is the ultimate goal of rehabilitation and perhaps the best defence against the risk factors for secondary stroke and hospital readmission [35]. Moreover, OT is the only spending category where additional spending has a statistically significant association with lower readmission rates, indicating a reduction in costs [36].

The available evidence is still limited; therefore, it is convenient to delve into the subject to determine the suitability of investment in OT modalities. Demonstrating whether the intervention of OT in the hospital discharge process has an impact on a patient’s functional recovery and prognosis, as well as its cost-effectiveness, could be an important contribution to the field. The objective of this review is to clarify the availability of the literature on OT intervention in the process of hospital discharge and its impact on stroke patients, caregivers, and the health system, which could have great significance for clinical and community-based occupational therapy practices, as well as healthcare delivery and policies. Therefore, our review question is “What is the evidence for interventions within the scope of occupational therapy to support post-stroke hospital to home discharge”?

## 2. Materials and Methods

### 2.1. Eligibility Criteria

A systematic search was carried out to search for available evidence related to the study topic and the PRISMA statement was followed [37]. Only studies published in the last 10 years from 1 January 2011 to 31 December 2021 and published in Spanish and English were selected. The type of study design accepted was RCT, only including those that investigated the benefits of interventions within the scope of OT for adults in the process of post-stroke hospital-to-home discharge. The studies had to carry out the intervention in the patient’s transition to home and evaluate the improvements in the functional ability of the person with stroke. Therefore, studies that carried out interventions for patients who were discharged to rehabilitation units or nursing homes were excluded.

#### Search Objective (PICO)

Population: Adults post-stroke.

Intervention: Interventions within the scope of occupational therapy to support discharge from an acute care hospital to home.

Comparison: Comparison of the results of those who received this intervention together with the usual care with those who only received the usual care. Effectiveness at 3, 6, and 12 months after the intervention.

Outcome: Impact of the intervention on the person with stroke, the caregiver, and the healthcare system.

### 2.2. Information Sources and Search Strategy

A search was performed in the following databases: Pubmed, Web of Science, PsycINFO, Scopus, Otseeker, and Dialnet. The keywords and descriptors used according to MeSH were *stroke rehabilitation* and *patient discharge* and a screening was carried out only accepting interventions within the scope of OT. The connector used was AND. The final search strategy was (patient discharge) AND (stroke rehabilitation). The search for all published studies was conducted in June 2020 and updated in January 2022.

The quality assessment of the selected studies was carried out by two independent researchers using the PEDro scale [38,39]. The PEDro scale is used to rate clinical trials included in systematic reviews in physiotherapy, health, and medical research. The scale contains 11 items that assess the presentation of the study’s statistical analysis and internal validity and its confidence interval is 95% = 0.57–0.76 (Maher, Sherrington, Herbert, Moseley, and Elkins, 2003). The scores of the studies are in Table 1. The mean score obtained according to the PEDro scale in assessing the methodological quality of the clinical trials and pilot studies was 8/10. The levels of evidence were also added to Table 1 using The Oxford Levels of Evidence [40].

### 2.3. Data Collection Process

The studies identified in the search were independently selected and blinded by two investigators. Disagreements were discussed and a third reviewer was consulted who assessed the eligibility of the study.

### 2.4. Risk of Bias Assessment

**Figure 1:**
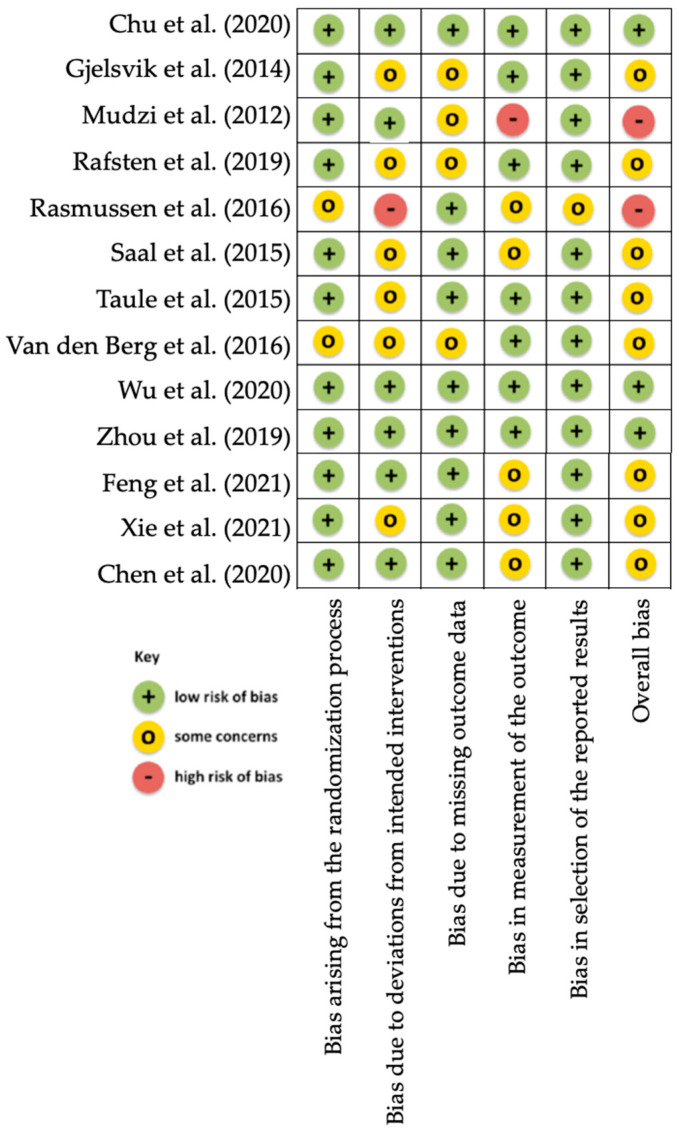
Risk of bias assessment according to the Cochrane Collaboration’s tool (RoB 2.0) for randomised controlled trials.

The levels of evidence from The Oxford Levels of Evidence were taken into account, finding a strong strength of evidence (Table 1) [40]. *Papers included were classified as 1B (individual RCT with narrow confidence intervals) and 2B (low-quality RCT due to <80% follow-up)*. The risk of bias assessment for the randomised controlled trials was carried out according to the Cochrane Collaboration’s tool (RoB 2.0) and showed a low risk of bias in 23% of the included studies, a moderate risk of bias in 62%, and a high risk of bias in the remaining 15% (Figure 1) [41].

**Table 1:** Characteristics of the included studies.

Authors and Year	Level of Evidence, Quality, and Risk of Bias	Participants, Inclusion Criteria, and Study Setting	Intervention and Control Groups	Intervention Details (Type, Session Duration and Frequency, and Duration of the Intervention) and Outcome Measures	Results
*Gjelsvik et al.* *(2014)* [42]	Level 2B PEDro: 8 Risk of bias: M	<7 days after stroke and 6–12 h after admission. NIHSS 2–26. Being discharged to home.	1. ESD at DC (52). 2. ESD at home (60). 3. Conventional treatment (55). Total N: 167.	Assessments: PASS, mRS, BI, NIHSS. Includes OT. 3 months of intervention.	Showed no difference in postural balance (3 m). Groups 1 and 2 were more effective in trunk control, gait perception, and ADLs.
https://doi.org/10.1136/bmjopen-2013-004358 (accessed on 20 June 2021).					
*Mudzi et al. (2012)* [43]	Level 2B PEDro: 5 Risk of bias: H	First stroke. Patient and caregiver.	1. Registration in training program (100). 2. Conventional treatment (100). Total N: 200.	Caregiver training program to increase the patient’s functional ability. It consists of 1 session of 45 min - 1 h (pre-discharge) and another session at 3 months. Evaluations: BI, RMI, at 3, 6, and 12 months.	Functional skills improved but not significantly.
https://doi.org/10.12968/ijtr.2012.19.7.380 (accessed on 20 June 2021).					
*Rafsten et al.* *(2019)* [44]	Level 1B PEDro: 9 Risk of bias: M	>18 years old. NIHSS 0-16. BI > 50. MoCA of <26 si. BI = 100. Address near the hospital.	1. Discharge with Very Early Supported Discharge, (VESD) (69). 2. Usual treatment (71). Total N: 140.	Treatment of OT, physiotherapy, and nursing. They set goals for the patient with pre-discharge COPM. It consists of 2-4 visits per week from OT / physiotherapy and 1-2 from nursing for 1 month. Evaluation at discharge and at 3 and 12 months.	Showed no difference in HADS-A. Significant improvement in mRS at 3 months but evened out at 12. VESD led to faster recovery.
https://doi.org/10.1186/s12883-019-1503-3 (accessed on 20 June 2021).					
*Rasmussen et al.* *(2016)* [45]	Level 1B PEDro: 8 Risk of bias: H	18 years old. mRS 0-3. Being discharged to home. Patient and caregiver.	1. Home discharge with rehabilitation (38). 2. Usual treatment (33). Total N: 71.	OT, physiotherapy, nursing, and medical treatment. A member of the team drives the patient home for rehabilitation; 1-3 times a week (as an inpatient) and 1-5 times a week (after discharge) for 1 month. Evaluation at 3 months.	Improved mRS and EuroQol-5D. Disability decreased and quality of life improved. Correlation of training minutes with BI, mRS, and MAS results. It proved to be cheaper than usual treatment.
https://doi.org/10.1177/0269215515575165 (accessed on 20 June 2021).					
*Saal et al. (2015)* [46]	Level 1B PEDro: 10 Risk of bias: M	>18 years old. First stroke. Address near the hospital.	1. Discharge within the stroke support program (130). 2. Conventional treatment (135). Total N: 265.	Home visits and calls. Individualised treatment. It consists of 6 educational sessions in 1 year + 12 contacts (phone, email, or face to face). Evaluation: SIS at discharge and 12 months.	Did not improve physical function, depression, or quality of life. Suggested a reduction in mortality risk.
https://doi.org/10.1179/1074935714Z.0000000047 (accessed on 20 June 2021).					
*Taule et al.* *(2015)* [47]	Level 2B PEDro: 9 Risk of bias: M	Admission to the program 6–12 h after the stroke. NIHSS 2-26. mRS 2-0. Being discharged to home.	1. ESD at DC (50). 2. ESD at home (53). 3. Conventional treatment (51). Total N: 154.	Treatment of OT and physiotherapy, individualised, at home, and in group in the DC. Evaluation: AMPS. The DC group received 22 hours in total. ESD group at home received 17 hours in total. Four-week intervention.	Taule et al. (2015) [47]
https://doi.org/10.3109/11038128.2015.1042403 (accessed on 20 June 2021).					
*Chu et al. (2020)* [48]	Level 1B PEDro: 9 Risk of bias: L	18–79 years old, mRS 3–5, and BI < 80. Patient must have a caregiver.	1. Discharge to home with a nurse-trained caregiver + calls for up to 8 weeks (31). 2. Usual treatment (30). Total N: 61.	Nursing treatment (with mobile app for scales), 3 times a week in hospital. Once discharged, telephone contact. Evaluation before discharge and at 3 and 6 months.	Significant differences at 6 months with BI. There were no differences in EQ-5D and CBI.
https://doi.org/10.1016/j.jstrokecerebrovasdis.2020.105382 (accessed on 20 June 2021).					
*Wu et al. (2020)* [49]	Level 1B PEDro: 9 Risk of bias: L	18–80 years old. NIHS 5–15, upper limb limitation, Brunnstrom II-III, being discharged to home, caregiver capacity scale < 40.	1. Home discharge with tele-rehabilitation (30). 2. Usual treatment (31). Total N: 61.	Treatment by neurologist, nurse, therapist, and counsellor + caregiver, establishing individualised goals. Treatment by video call 2 times a week. Evaluation before discharge and at 1 month, 2 months, and 3 months after discharge.	Safe and efficient program to promote motor functionality, ADL, and quality of life.
https://doi.org/10.1016/j.jstrokecerebrovasdis.2020.105328 (accessed on 20 June 2021).					
*Zhou et al.* *(2019)* [50]	Level 1B PEDro: 9 Risk of bias: L	18–79 years old. BI < 80.	1. Discharge with program support (116). 2. Conventional treatment 128). Total N: 244.	Smartphone application. Pre-discharge stroke training and nurse education. Sessions of 15–30 min + 3 support calls (2, 4, and 8 weeks after discharge). Evaluation at 3 and 6 months (BI).	There was no significant improvement in ADLs.
https://doi.org/10.1161/STROKEAHA.118.021558 (accessed on 20 June 2021).					
*Feng et al.* *(2021)* [51]	Level 1B PEDro: 7 Risk of bias: M	Diagnosis of stroke, >60 years old, with a family member, and who has no cognitive impairment.	1. Control group (n = 60). 2. Intervention group (n = 60). Total N: 120.	An integrated intervention group was established. An intervention plan was formulated and implemented and there was a patient bedside visit to clarify that the patient’s condition was being managed. After discharge, patients received phone and WeChat follow-ups and home visits once a month.	Hospital-integrated service model (HCISM) improved self-care and self-efficacy of stroke patients and medical compliance behaviour and reduced negative emotions.
https://dx.doi.org/10.21037/apm-21-602 (accessed on 15 January 2022).					
*Chen et al.* *(2020)* [52]	Level 1B PEDro: 8 Risk of bias: M	Diagnosis of stroke with lower limb spasticity and ability to follow instructions.	1. Intervention group (n = 59). 2. Control group (n = 62). Total N: 121.	Intervention group participated in HREPro (individually tailored, year-long rehabilitation intervention program conducted at home by a nurse who received therapy training). Evaluation at 3, 6, and 12 months.	HREPro was beneficial in patients’ lower limb spasticity post-stroke by promoting the recovery of motor function, reducing muscle spasticity, improving walking ability, and enhancing ADL.
https://doi.org/10.1016/j.anr.2020.08.007 (accessed on 15 January 2022).					
*Xie et al.* *(2021)* [53]	Level 1B PEDro: 8 Risk of bias: M	Diagnostic criteria of cerebral infarction, 40–80 years, Abbreviate Mental Score > 7.	1. Discharge with rapid rehabilitation nursing (n = 68). 2. Routine nursing (n = 68) Total N: 136.	Intervention group was given rapid rehabilitation nursing and follow-up.	Rapid rehabilitation nursing combined with continuous nursing promoted the rapid recovery of patients with stroke, which improved motor function, reduced unhealthy psychology, and improved quality of life.
https://doi.org/10.1155/2021/8065868 (accessed on 15 January 2022).					
*Van den Berg et al. (2016)* [54]	Level 2B PEDro: 7 Risk of bias: M	24h-3 months after discharge. Functional Ambulation Category < 5. MMSE > 18. No depression.	1. Registration in training program (31). 2. Conventional treatment (32). Total N: 63.	Training program with the help of the caregiver (tele-rehabilitation using app for tablet). Sessions of 30 min 5 times a week for 8 weeks. Evaluation at 8 and 12 weeks (SIS).	Caregiver fatigue decreased and self-efficacy increased at week 12. Mobility and IADL (weeks 8 and 12) improved and readmissions decreased in the first year.
https://doi.org/10.1161/STROKEAHA.116.013431 (accessed on 20 June 2021).					

Note: H = High; M = Moderate; L = Low; mRS = modified Rankin Scale; BI = Barthel Index; EQ-5D = European Quality of Life-5 Dimensions; CBI = Caregiver Burden Index; NIHSS = National Institute of Health Stroke Scale; ESD = Early Supported Discharge; DC = Day Center; PASS = Postural Assessment Scale for Stroke patients; OT = Occupational Therapy; ADL = Activities of Daily Living; RMI = Rivermead Mobility Index; MoCA = Montreal Cognitive Assessment; VESD = Very Early Supported discharge; COPM = Canadian Occupational Performance Measure; HADS = Hospital Anxiety and Depression Scale; EuroQoL-5D = European Quality of Life-5 Dimensions; MAS = Motor Assessment Scale; SIS = Stroke Impact Scale; AMPS = Assessment of Motor and Process Skills; MMSE = Mini-Mental State Examination; IADL = Instrumental Activities of Daily Living.

## 3. Results

### 3.1. Information Sources Selection

**Figure 2:**
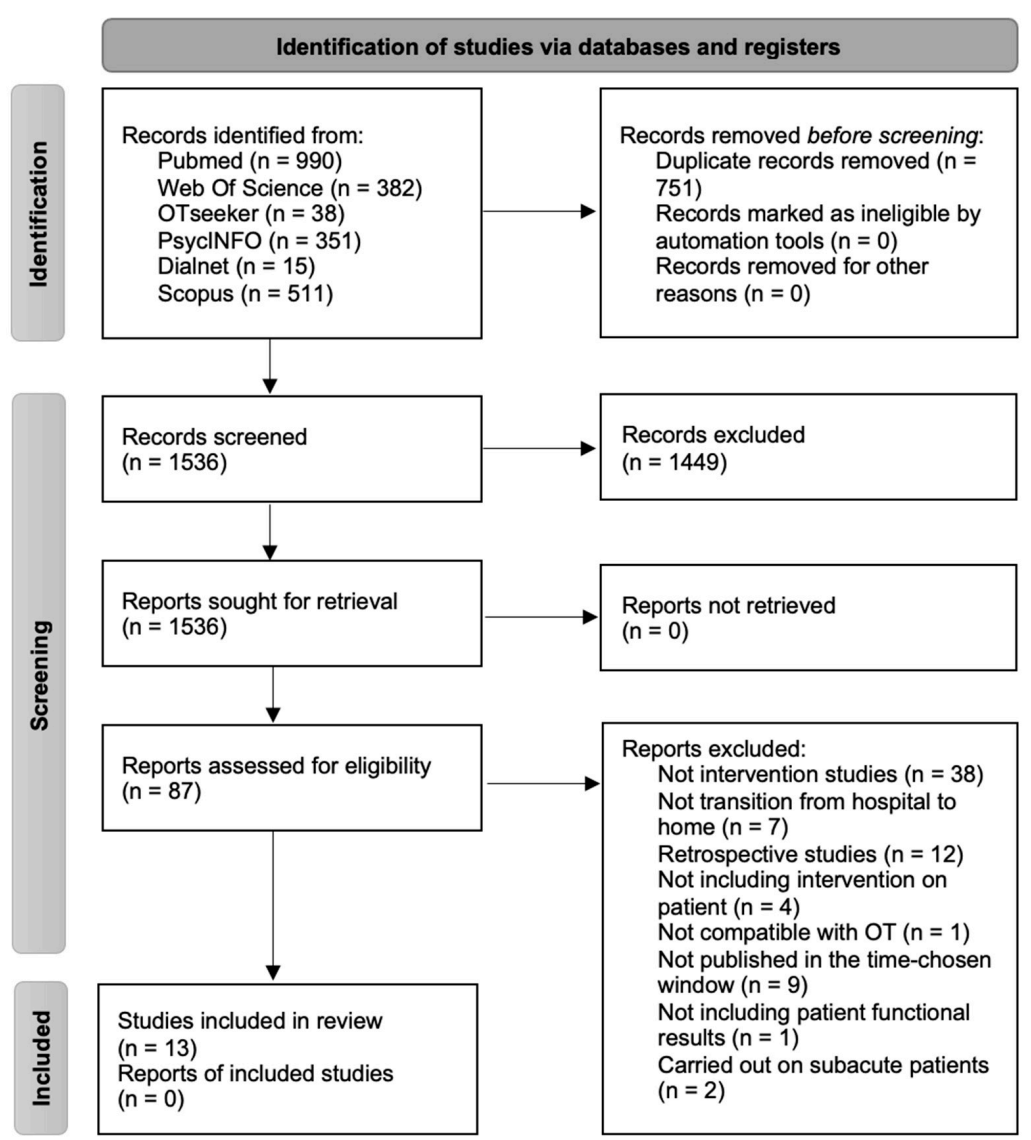
PRISMA 2020 flow diagram.

A systematic search was carried out in Pubmed, Web of Science, PsycINFO, Scopus, Otseeker, and Dialnet, finding 2285 studies. After applying the exclusion criteria, we obtained a total of 13 articles, all of them published in English. The information flow is presented in the PRISMA diagram (Figure 2) [37].

### 3.2. Study Characteristics

Table 1 shows the main author and year of publication, level of evidence, risk of bias, RCT quality, profile of participants, study groups, number of participants, details of the intervention, and the most significant results.

#### 3.2.1. Population

The reviewed studies focused only on the adult population with a diagnosis of stroke, who started the intervention in hospital, and who were discharged directly to their homes.

#### 3.2.2. Year and Country of the Studies

In the last 10 years, research on stroke patients has been a popular topic; however, studies that also include an OT approach are very limited. Of the 13 studies included in the review, 6 of them included the role of the occupational therapist and were conducted in Denmark, Sweden, Germany, Norway, and South Africa, highlighting the resurgent importance of occupational therapy in Northern European countries [42,43,44,45,46,47]. However, of the seven included studies that did not name OT, six of them were conducted in China [48,49,50,51,52,53] and one in Australia [54]. The year of publication of the studies varies, with 2016 the year when more studies were published (three of them), followed by 2019, 2020, and 2021, with two studies published in each of these years.

#### 3.2.3. Size of the Study Object Samples

All of the investigations were carried out on the adult population, with three of them exceeding 200 participants [43,46,50].

#### 3.2.4. Age Characteristics and Other Conditions of the Studied Participants

This systematic review focused on the adult population and mostly included studies focusing on people aged 18–80. Regarding the gender of the participants, 53% were men (n = 955) and 49% were women (n = 848), with an approximate average age of 67. Occupational therapy in stroke involves working with the patient, the caregiver, and the family unit. All of the interventions focused on improvements in the functional ability of the person with stroke (n = 1803), whereas the caregiver was considered an important factor in patient rehabilitation in 85% of studies (the study included, for example, the psychoeducation of the caregiver with the aim of improving knowledge, skills, and confidence) [43,44,45,46,48,49,50,51,52,53,54].

### 3.3. Intervention

The included studies investigated the effectiveness of the following interventions in the discharge process of stroke patients:

1. Coordination in the process of transition from hospital to home [42,45,47,48,50,51].
2. Home evaluation and adaptation [45,52].
3. ADL training [45,47,48].
4. Promotion of participation in meaningful activities [44,47].
5. Task-oriented training approach [42,43,49,54].
6. Contribution of stroke information manuals or written information [46,50].
7. Support through professional guidance and useful techniques [45,47,50].
8. CD with daily training [50].
9. Training program app for the caregiver [48,54].
10. Exercises to improve gait and mobility [52,53,54].
11. Video conferencing [49,54].
12. Use of activity monitors (Fitbit Zip) [54].
13. Functional rehabilitation [44].
14. Recommendations, guidance, and counselling [44,45,46,48,49,50,51,52,53].
15. Stroke prevention [45,46].
16. Information on referrals to other therapeutic services [44,45,49,51].
17. Educational sessions [46].
18. Training for the patient and caregiver (mobilisation and manipulation techniques, postural care, transfers, continence, ADL assistance and communication, prevention of bedsores, positioning, walking facilitation, going down and up the stairs, use of the bathroom, personal care, mobility in bed, and sexuality) [43,48,53].
19. Recommendation and use of technical aids [45].
20. WeChat group for disabled patients with stroke [51].
21. Meaningful conversations to stimulate emotions and language functions [53].

#### Instruments Used in the Included Studies

Standardised assessments were the most frequently used instruments to carry out the functional assessments (Table 2). As can be seen, the most widely used evaluations were the Barthel Index, used in 77% of the studies, and the modified Rankin Scale, used in 40% of them. The results are compared in Table 2 and highlight the significant outcomes of the instruments used at one, three, and six months and one year after the stroke.

### 3.4. Comparison

In this study, a comparison was made of the results of those who received the intervention together with usual care and those who only received the usual care, taking into account the effectiveness at 1, 3, 6, and 12 months after the intervention. It is noteworthy that 8 out of the 13 included studies focused on comparing the results three months after the stroke, thus obtaining more significant data at this time of the intervention (Table 2).

### 3.5. Outcomes

Table 2 highlights the significant values resulting from the comparison of the experimental and control groups, thus showing the possible benefits of the interventions. From the 13 studies included in the review, the authors measured the effectiveness of 21 interventions within the scope of OT on people with stroke. However, not all studies obtained significant values.

#### 3.5.1. Improvements in Functional Ability

Table 1 and Table 2 show the characteristics of the studies included in the systematic review as well as the type of evaluations carried out. All of the studies compared the experimental group that included an intervention in the transition from hospital to home with conventional care, although two of them included a third study group (an intervention program in a Day Centre).

Regarding the improvements in functional ability results, 69% of the reviewed studies showed a significant improvement at three months, 30% of them at six months, and 15% after one year. Therefore, 92% of the studies showed a significant improvement when comparing the control and intervention groups in some of the evaluations dedicated to measuring the improvements in the functional ability of the patient. Only one study failed to prove any significant difference; however, it suggested a reduction in the risk of patient mortality [46].

In general, this type of intervention that aims to promote the independence of the person with stroke translates into an increase in rehabilitation time of up to 1000 min, which could lead to an improvement in the patient’s functional skills [50]. Despite these positive results, the included studies generally concluded with the need for further studies to prove the efficacy of the program, except for three of them that concluded by validating the safety and efficiency of the intervention for the promotion of a healthy rehabilitation and quality of life [49,51,53].

#### 3.5.2. Caregiver Experience

Despite the fact that most studies (85%) included the caregiver in the intervention, none of these studies obtained a significant difference in the standardised evaluations regarding their involvement. A notable concern of the therapists in the included studies was the possible inconsistencies in the provision of care by caregivers, as well as the patients’ reluctance to exercise without adequate supervision.

In one of the studies, the caregivers in the intervention group reported higher self-efficacy as measured by the General Self-Efficacy Scale (−3.9, 95% CI−6.7 to 0.0; *p* = 0.0072) at week 12, with a trend toward lower levels of fatigue (4.8, 95% CI−0.1 to 9.8; *p* = 0.0543), anxiety, and depression (2.9, 95% CI−0.1 to 5.8; *p* = 0.0555) [50]. However, these differences were not statistically significant. Significant improvements in caregiver self-efficacy and fatigue were also seen at week 12, but not week 8, in both the intention-to-treat and per-protocol analyses, suggesting that it is important to involve caregivers in the hospitalisation phase, but that the intervention takes some time to impact the caregiver’s outcomes. In another study, the patients belonging to the experimental group improved their RMI scores by a mean score of 0.7, which was moderately significant from a clinical point of view, leading to the belief that perhaps patients cared for by trained caregivers exercised slightly more and this could be attributed to greater confidence in the mobilisation process [43]. Therefore, the included studies did not show significant results in terms of caregiver burden, anxiety, or depression. Nevertheless, several of them reported that the presence of a caregiver in the process influenced the patient’s adherence to treatment and their consequent completion of the study.

#### 3.5.3. Effectiveness of the Program in Terms of Time and Cost

Not all the studies reviewed included data on lengths of hospital stays, readmissions, or costs related to stroke treatment. However, one of them, conducted in Denmark, in addition to proving positive and significant results in mRS, EQol-5D, MAS, and BMI at 3 months of intervention, included economic data showing a reduction of 0.2% in costs in favour of home intervention programs [45]. Moreover, it referred to a recent systematic review that related ESD to cost savings in all six included studies, as well as other studies that have found savings of up to 20% compared to the usual care [55,56,57,58]. Another one of the included studies stated that the program carried out did not increase costs but neither could they prove a substantial and significant cost saving [44]. Finally, a third article included in the review showed an important reduction in readmissions in the intervention group (*p* value 0.0432), which translates into long-term cost savings [54].

## 4. Discussion

In this systematic review, studies published in the last ten years on support for stroke patients during the hospital-to-home discharge process have been reviewed in order to illustrate the impact of different interventions within the scope of OT in this process, especially in terms of the improvements in the functional ability of the person with stroke, the experience of the caregiver, and their consequent impact on the health system. The lack of specific studies of OT in this field suggests the neglect of OT in the context of early stroke rehabilitation and hospital discharge.

The limitations observed in the included studies were the small sample size, lack of registration of treatment received by the participants in the control group, possible bias caused by the use of different testers in pre-post assessments and participants being aware of their group allocations, inconsistency in the delivery of care by caregivers, lack of blinded investigators, and the fact that single-centre studies can only represent the circumstances of regional patients. In a study carried out in China, both the nursing staff and the caregivers participating in the study reported difficulties in acquiring the necessary skills to carry out rehabilitation, which makes us consider the need for specialised staff, as well as offering the most complete support to the patient’s caregiver to avoid feelings of inadequacy and ineffectiveness [50].

Among the main strengths of the studies, we found that they included pragmatic interventions that begin in coordination with the hospital stroke unit and end in a natural context for the patient with individualised treatment goals. OT practitioners working with post-stroke adults must understand the implications of their patients’ limitations on occupational performance and choose individualised interventions based on clinical reasoning and available scientific evidence. The studies agreed that individual planning and rehabilitation should start before discharge, which could lead to a more rapid independent recovery. Furthermore, despite the fact that they were unable able to give clear recommendations on the best type of program for stroke survivors in the transition from hospital to home, they concluded that patients with both social and physical needs could benefit from multidisciplinary services such as ESD.

Regarding the evaluations used by the selected studies, standardised assessments were the most frequently used instruments, with the Barthel Index the most widely used evaluation followed by the modified Rankin Scale. According to the interventions, the caregiver was involved in most of them. It is important to mention that all the studies included prioritized, individualised objectives for the patient, which made each treatment unique and therefore difficult to compare due to their heterogeneity. The study by Taule et al. carried out in Norway, did not find the expected significant differences in terms of improvements in patients’ functional abilities and they discussed the possibility that this was due to the national guidelines’ high-quality rehabilitation requirements [47]. Another study referred to the possibility that spontaneous recovery combined with generally high baseline scores that implied a ceiling effect, could have affected the results [42].

An important fact to highlight is that most studies focused on specific populations, with the majority being carried out in northern Europe [42,44,45,46,47] and China [48,49,50,51,52,53]. Therefore, conducting studies in other geographical settings would be of interest to validate this type of intervention in different contexts and check whether its effectiveness can thus be separated from the particulars of the environment. It is also worth mentioning the limited evidence published in the period before and during the COVID-19 pandemic.

Although the scientific evidence published on this topic has increased in recent years, further studies are necessary to confirm the benefits of the associations between different treatments to improvements in the patient’s functional ability. The results have shown that home rehabilitation programs can improve the patient’s autonomy, however, these studies included other figures besides the occupational therapist, who can be an important factor in the improvement of the patient, such as physiotherapists, nurses, neurologists, and counsellors [44,45,47,49,51]. Therefore, it is not possible to draw reliable conclusions, but it is a reminder of the need for further research, which must be conducted to identify the best practices for OT discharge planning and evaluate their efficiency and cost-effectiveness.

## 5. Conclusions

Overall, the literature addressing interventions within the scope of OT practices for people going home from hospital post-stroke is limited as we found when carrying out this systematic search, thus demonstrating a clear need to obtain more evidence. 

Although most of the studies concluded that early interventions carried out in the process of hospital discharges were effective in terms of patients’ functional recoveries, as well as led to improvements in self-efficacy and a reduction in fatigue in caregivers, the heterogeneity of the interventions and the variety of measurements and timelines precludes us from drawing specific conclusions. Stroke discharges and rehabilitation plans are carried out in a multidisciplinary manner, making it difficult to evaluate the extent to which OT contributed to the functional recovery of patients, especially when the studies included were undertaken with combined interventions. In the accepted literature, stroke patients were not only treated with OT before discharge, therefore the evidence cannot support the effect of OT intervention on functional recovery before discharge. It is necessary to carry out further research to evaluate early OT intervention in the process of hospital discharge and study its influence on the person with stroke and its cost-effectiveness. 

In conclusion, more studies are needed to prove the efficacy of occupational therapy interventions in post-stroke patients, as well as their impact on the health system.

## Figures and Tables

**Table 2:** Instruments used for evaluations and significant values.

Studies	Instruments	Significant Results (*p* Value)			
		1 Month	3 Months	6 Months	1 Year
Gjelsvik et al. (2014) [42]	PASS **TIS-modNV** - **Trunk impairment** **NRS** - ADL FAC TUG 5mTW		0.044 0.016		
Mudzi et al. (2012) [43]	**BI** RMI Mobility		0.01		
Rafsten et al. (2019) [44]	BI **HADS-A** HADS-B **mRS** MoCA		0.05 <0.01		
Rasmussen et al. (2016) [45]	Length of stay in hospital BMI **mRS** **Modified BI** - **ADL index scores** **MAS** CT-50 **EuroQol–5D** **Costs**		<0.00001 <0.00001 <0.001 0.01		Savings of 0.2%
Saal et al. (2015) [46]	SIS - Physical subscale WHOQOL-BREF GDS SCL-90-R				
Taule et al. (2015) [47]	**AMPS** - **Motor scale** - **Process scale** - **Motor cut-off** - **Process cut-off** **mRS**		<0.001 <0.001 <0.001 <0.001 0.03		
Chu et al. (2020) [48]	**BI** EQ-5D CBI			0.0312	
Wu et al. (2020) [49]	**FMA** **BBS** **TUG** **6mWT** **Modified BI** **SSQol** - **Energy** - **Family** - **Mobility** - **Self-care** - **Social role** **Work**	<0.001	<0.001 <0.001 <0.001 <0.001 <0.001 <0.001 <0.001 <0.001 <0.001 <0.001 <0.001 <0.001		
Zhou et al. (2019) [50]	BI **FAC** mRS PHQ-9 EQ-5D CBI Expenses in hospital Length of stay in hospital			0.04	
Feng et al. (2021) [51]	**Modified BI** **GSES** **SAS** **SDS**		0.000 0.000 0.000 0.000		
Chen et al. (2020) [52]	**FMA** **MAS** 10-Meter walk test - **Gait Speed** - **Step Size** **BI**		<0.001 0.033	<0.001 0.031 0.042 0.032 <0.001	<0.001 <0.001 <0.001 <0.001 <0.001
Xie et al. (2021) [53]	**Nursing Effficiency** **Modified BI** **MAS** **SAS** **SDS** **QLI**			0.033 <0.05 <0.05 <0.05 <0.05 <0.05	
Van den Berg et al. (2016) [54]	**SIS** - **Communication** - **Memory** Length of stay in hospital RMI BI Nottingham Extended ADL **TUG** mRS Length of stay in hospital Readmissions **Hospital anxiety and depression scale** General self-efficacy scale FSS **CarerQOL** EC strain index	2 months 0.0179 0.0118 0.0072	0.0246 0.0003 0.0319		0.0464

Note: AMPS = Assessment of Motor and Process Skills; mRS = modified Rankin Scale; PASS = Postural Assessment Scale for Stroke patients; TIS-modNV = modified Trunk Impairment Scale Norwegian Version; NRS = Numeric Rating Scales; FAC = Functional Ambulation Categories; TUG = Timed Up-and-Go; 5mTW = 5 m timed walk; BI = Barthel index; PHQ-9 = Patient Health Questionnaire 9; EQ-5D = European Quality of Life-5 Dimensions; CBI = Caregiver Burden Index; SIS = Stroke Impact Scale; RMI = Rivermead Mobility Index; Nottingham Extended ADL = Nottingham Extended Activities of Daily Living; FSS = Fatigue Severity Scale; CarerQOL = Care-Related Quality Of Life; EC strain index = Expanded Caregiver strain index; WHOQOL-BREF = World Health Organization Quality of Life questionnaire; GDS = Global Deterioration Scale; SCL-90-R = The Symptom Checklist-90-Revised instrument; HADS = Hospital Anxiety and Depression Scale; MoCA = Montreal Cognitive Assessment; BMI = Body Mass Index; MAS = Motor Assessment Scale; CT-50 = Cognitive Test-50; FMA = Fugl–Meyer Assessment; BBS = Berg Balance Scale; 6mWT = 6 m timed walk; SSQol = Stroke Specific Quality of Life scale; GSES = General Self-Efficacy Scale; SAS = Zung’s Self-Rating Anxiety Scale; SDA = Zung’s Self-rating Depression Scale; QLI = Quality of Life Index.

## Data Availability

Not applicable.
